# MMP9 Processing of HSPB1 Regulates Tumor Progression

**DOI:** 10.1371/journal.pone.0085509

**Published:** 2014-01-20

**Authors:** Seo-hyun Choi, Hae-June Lee, Yeung Bae Jin, Junho Jang, Ga-Young Kang, Minyoung Lee, Chun-Ho Kim, Joon Kim, Sam S. Yoon, Yun-Sil Lee, Yoon-Jin Lee

**Affiliations:** 1 Division of Radiation Effects, Korea Institute of Radiological & Medical Sciences, Seoul, Korea; 2 School of Life Sciences and Biotechnology, Korea University, Seoul, Korea; 3 College of Pharmacy & Division of Life and Pharmaceutical Sciences, Ewha Womans University, Seoul, Korea; 4 Laboratory of Tissue Engineering, Korea Institute of Radiological & Medical Sciences, Seoul, Korea; 5 Department of Surgery, Massachusetts General Hospital and Harvard Medical School, Boston, Massachusetts, United States of America; Medical College of Wisconsin, United States of America

## Abstract

Matrix metalloproteinases regulate pathophysiological events by processing matrix proteins and secreted proteins. Previously, we demonstrated that soluble heat shock protein B1 (HSPB1) is released primarily from endothelial cells (ECs) and regulates angiogenesis via direct interaction with vascular endothelial growth factor (VEGF). Here we report that MMP9 can cleave HSPB1 and release anti-angiogenic fragments, which play a key role in tumorprogression. We mapped the cleavage sites and explored their physiological relevance during these processing events. HSPB1 cleavage by MMP9 inhibited VEGF-induced ECs activation and the C-terminal HSPB1 fragment exhibited more interaction with VEGF than did full-length HSPB1. HSPB1 cleavage occurs during B16F10 lung progression in wild-type mice. Also, intact HSPB1 was more detected on tumor endothelium of MMP9 null mice than wild type mice. Finally, we confirmed that secretion of C-terminal HSPB1 fragment was significantly inhibited lung and liver tumor progression of B16F10 melanoma cells and lung tumor progression of CT26 colon carcinoma cells, compared to full-length HSPB1. These data suggest that *in vivo* MMP9-mediated processing of HSPB1 acts to regulate VEGF-induced ECs activation for tumor progression, releasing anti-angiogenic HSPB1 fragments. Moreover, these findings potentially explain an anti-target effect for the failure of MMP inhibitors in clinical trials, suggesting that MMP inhibitors may have pro-tumorigenic effects by reducing HSPB1 fragmentation.

## Introduction

Heat shock proteins (HSPs) were first identified as molecular chaperones; recently, however, additional functions of HSPs in physiological and pathological processes have been elucidated [1]. Small HSPs function in apoptosis inhibition, cellular protection, and proteosomal degradation through direct binding of various molecules [2], [3]. HSPB1 (HSP27/25 in the human/mouse form) and αB-crystallin, both members of a small HSP protein family, share the conserved domain of α-crystallin. It has recently been reported that the N-terminal domain serves as a platform for modulation of HSPB1 assembly and dynamics and consequently regulates their function [4].

Serum HSPB1 has been suggested as a prognostic marker of tumor malignancy because HSPB1 serum levels are elevated in cancer patients [5], [6], [7]. We have previously shown that overexpression of HSPB1 in cancer cells induces radio- and chemo-resistance [8], [9]. However, the role of HSPB1 in cancer remains unclear. HSPB1 serine 82 phosphorylation by VEGF activation of PKC-mediated PKD induces endothelial migration and tubulogenesis, indicating the potential importance of HSPB1 in VEGF-dependent angiogenesis [10]. Recently, we reported that extracellular HSPB1, secreted primarily from endothelial cells, maintains the angiogenic balance via direct binding with VEGF [11].

The matrix metalloproteinases (MMPs) have been associated with multiple pathophysiological processes such as arthritis, cancer, atherosclerosis, tissue ulcer and fibrosis. Moreover, the roles of MMPs in cancer progression are thought to be more complex than their degradative action on ECM components [12], [13]. Overexpression of some members of MMPs has been correlated with the invasion, metastasis, and poor prognosis [14]. For examples, MMP2, membrane type-1 MMP (MT1-MMP), and MMP9 are implicated in the invasion and metastasis in head and neck squamous cell carcinoma [14]. Knockout of MMP-9 leads to reduced skin and pancreatic carcinogenesis and metastasis showing delayed tumor vascularization [15]. Otherwise, knockout of MMPs −3, −8, and −9 leads to enhanced tumorigenesis and metastasis in some animal models of cancer [16].

In addition, MMP proteolysis regulates extracellular environment homeostasis, including mechanisms of host-resistance to tumors [17]. Proteolytic processing of some ECM substrates, such as laminin 5, releases new molecules with properties distinct from their precursor protein [18]. Angiostatin, an anti-angiogenesis blocker, is a product of plasminogen formed by MMP2, MMP3, MMP7, MMP9, and MMP12 cleavage *in vitro*. Endostatin, an endogenous anti-angiogenic molecule, is generated from type XVIII collagen by MMP3, MMP9, MMP12, MMP13, and MMP20 processing *in vitro* [18]. Because inhibition of some MMPs may have pro-tumorigenic effects (making them anti-targets), drug selectivity must be considered to distinguish the actions of proteases that contribute to tumor progression from those that are crucial for host defense [16].

In the development of MMP inhibitors (MMPI) as anticancer drugs, extensive phase III clinical trials in patients with advanced cancer failed. Universally, the trials failed to increase survival [19]. Therefore, for successful cancer therapy, MMPI must be developed to minimize adverse reactions as anti-targets.

In this study, we demonstrate that HSPB1 processing by MMP9 releases anti-angiogenic fragments of HSPB1 and this cleavage acts to maintain the angiogenic balance in tumor progression. Therefore, we suggest that MMP9-induced HSPB1 cleavage is an anti-target effect of MMPI that must also be considered in MMP target validation and MMPI drug development.

## Materials and Methods

### Cell Lines

HUVECs (human umbilical vein ECs) and HUVEC-Ls (human lung microvascular ECs) were obtained from Lonza. All ECs were used within nine passages. B6F10 melanoma and CT26 colon carcinoma cells were a kind gift from Sam S. Yoon. Human osteosarcoma cells (HOS) and COS-7 cells (monkey kidney fibroblast cells) were purchased from ATCC.

### Primary Tumor Model

k-ras*^LSL-G12D/WT^*; p53*^Flox/Flox^* mice, a gift from Sandra Ryeom, have been described previously [20], [21]. Soft tissue sarcomas were generated via intramuscular injection of Adeno-CRE (Cell Biolabs). Lung adenocarcinomas were generated via intranasal administration of Adeno-CRE. *In vivo* tumor model.

All protocols involving mice were approved by the Institutional Animal Care and Use Committee of the Korea Institute of Radiological and Medical Sciences. To generate lung tumor, 5× 10^5^–1.0×10^6^ B16F10 cells or CT26 cells were injected intrasplenically or into the tail vein of MMP9-null mutant mice or wild-type mice (C57BL/6 background). MMP9-null mice were purchased from the Jackson Laboratory. Six mice were used in each group. Lungs and livers were harvested two weeks after adenovirus treatment, weighed, and fixed in formalin.

### Cleavage Assays

Recombinant HSPB1 (rHSPB1) protein (2 µg; Stressgene) and wild-type or mutant HSPB1 proteins were purified using an anti-Flag M2 affinity gel. Purified proteins were then incubated for 5, 10, or 30 min at 37°C with MMPs (50 ng/ml) in a buffer containing 50 mM Tris (pH 7.4), 10 mM CaCl_2_, and 80 mM NaCl in a maximum volume of 60 µL. The reaction was terminated by addition of Laemmli buffer and resolved on a 10% gradient SDS-PAGE gel.

### Enzyme-Linked Immunosorbent Assay (ELISA)

Ninety-six well plates were coated overnight with Flag protein or N-terminal HSPB1 protein at 4°C. Serum was incubated for 1 hr at room temperature and then washed. The Myc antibody or C-terminal HSPB1 antibody were used as the primary antibodies. For detection of intact HSPB1 in mouse serum, the appropriate secondary antibodies were used. The assay was performed in accordance with the manufacturer’s instructions.

### VEGF-Binding Assay

VEGF _165_ was added to 96-well plates. The plates were incubated with 100 µL/well of serial dilutions of Flag-tagged HSPB1 wild type or C-terminal HSPB1 proteins in PBS plus 1% BSA at 37°C for 2 hours. The remaining proteins on VEGF were detected with an anti-flag monoclonal antibody (1∶1000, Sigma) using ELISA.

### Mass Spectrometry and Liquid Chromatography Mass Spectrometry

HSPB1 digested with MMP9 was separated by 15% SDS-PAGE. Bands of interest were excised, digested with trypsin, and analyzed by matrix-assisted laser desorption/ionization-time of flight mass spectrometry (MALDI-TOF MS) and liquid chromatography-mass spectrometry (LC-MS) peptide mapping. Also, the intact molecular weight was determined using MALDI-TOF MS and LC-MS. MALDI-TOF MS and LC-MS peptide mapping were analyzed by PROTEINWORKS Co. Ltd.

### Vector Constructs

The PPTLS control vector for secretion was made by insertion of a synthesized signal sequence (PPTLS) into the pcDNA3.1/myc-His A vector (Invitrogen) using BamHI and EcoRI sites [22]. Human HSPB1 or its deletion mutants were cloned into the expression vector p3xFLAG-myc-CMV^26^ (Sigma). Flag-tagged HSPB1 constructs were subcloned into the PPTLS control vector using EcoRI and XbaI sites. Lentiviral constructs for short hairpin RNAs (shRNAs) to silence human MMP9 and control lentiviral constructs were purchased from Santa Cruz Biotechnology.

### Purification of Recombinant HSPB1 Protein

To obtain recombinant HSPB1 and HSPB1 deletion mutant proteins, COS-7 cells were stably transfected with HSPB1 and HSPB1 deletion mutant constructs with the secretory signal sequence. For high-yield recombinant protein production, the BD CELLine 1000 system (BD Biosciences) was used, in accordance with the manufacturer’s instructions. The recombinant proteins were purified at 4°C by batch absorption using an anti-Flag M2 affinity gel (Sigma), again according to the manufacturer’s instructions.

### Immunoprecipitation and Immunoblotting

Recombinant Human VEGF-A_165_ (100 ng/mL; R&D Systems) was incubated for 6 h at 4°C with Flag-tagged HSPB1 (1 ug/mL) or HSPB1 fragment protein (1 ug/mL) in binding buffer containing 10 mM HEPES, 150 mM NaCl, 20 µug/mL BSA, and heparin (10 units). Flag antibodies with protein A-Sepharose (80 uL; Sigma) were incubated for 4 h at 4°C. The immune complexes were washed and analyzed by SDS-PAGE. Immunoblotting was performed using the Flag (Sigma) and VEGF (R&D Systems) antibodies.

### Immunofluorescence

Mice were euthanized and tissues were harvested and fixed in formalin for the preparation of paraffin sections. Paraffin-embedded tissue sections were deparaffinized in xylene, 95, 90, and 70% ethanol, followed by phosphate buffered saline (PBS). Epitopes were unmasked with 20 µg/mL proteinase K in PBS with 0.1% Triton X-100. Sections were co-immunostained overnight at 4°C with the CD31 mAb (1∶100; Santa Cruz) and the HSPB1 mAb (1∶1000; Santa Cruz), followed by incubation for 1 hr at room temperature with donkey anti-mouse Alexa 488- or anti-goat Alexa594-conjugated secondary antibodies (1∶500; Molecular Probes). The HSPB1 epitope for detecting the N-terminus (Abcam, ab5579) corresponds to amino acids 10–21 and the epitope for the C-terminus (Abcam, ab26942) corresponds to amino acids 145–205. Cell nuclei were labeled with Hoechst dye (1 ug/mL). Images were obtained with a Zeiss microscope. Microvessel density was quantified after CD31 immunostaining of five sections per tissue and five mice per cohort, as described previously. Additional immunohistochemical analyses of HSPB1 and CD31 were performed as described previously [23]. Images were obtained with a Zeiss microscope.

### Tumor-derived EC Isolations

Tumor-derived EC isolations were performed according to protocols described previously [11]. Tumor cells were grown in Dulbecco’s modified Eagle medium (DMEM).

### *In vitro* Endothelial Cell Assays

EC proliferation was assessed by MTT (3-(4, 5-dimethylthiazol-2-yl)-2, 5-diphenyltetrazolium bromide) assay, as described previously [11]. Recombinant human VEGF_165_ (30 ng/mL; R&D Systems) and purified HSPB1 wild-type or mutant proteins (1 ug/mL) were added where indicated.

### Tumor-transendothelial Cell Migration Assay

Transendothelial cell migration assays were performed using an assay kit (CytoSelect™ Tumor Transendothelial Migration Assay Kit; Cell Biolabs). HUVECs were plated onto 8-µm pore size inserts in 24-well plates and allowed to form a confluent monolayer on the membrane for 72 hrs. A confluent monolayer was checked by immunofluorescence-staining ECs with anti-CD31 antibody. After HOS cells were marked with CytoTracker, they were plated onto the endothelial cell monolayer in the upper inserts via bottom inserts with or without recombinant HSPB1 protein (1 ug/mL; Stressgene) or neutralizing HSPB1 antibodies (1 ug/mL; Stressgene). The tumor cells were allowed to transmigrate through the endothelium and membrane for 16 hrs. Non-migratory cells were removed and migratory cells were lysed. Fluorescence was determined with a fluorescence plate reader at 480 nm/520 nm.

### Statistical Analyses

Student’s *t*-test and an analysis of variance (ANOVA) were used to determine statistically significant differences between the experimental groups. Statistical analyses were performed using GraphPad Prism ver. 5.0 (GraphPad Software, Inc.). The band intensities of all immunoblots were quantified by volume densitometry using the Quantity-One software of the Bio-Rad imaging system.

## Results

### Soluble HSPB1 is Secreted by Tumor Endothelial Cells

HSPB1 is elevated in the serum of patients with many types of cancer, although its exact role in tumorigenesis and metastasis has yet to be determined [24], [25], [26]. Recently, we reported that soluble HSPB1 is released primarily from endothelial cells (ECs) [11]. To investigate the effect of soluble HSPB1 on tumor progression, we performed tumor cell transendothelial migration assays. Exogenous administration of HSPB1 inhibited the migration of HOS tumor cells through HUVECs, while HSPB1-neutralizing antibodies that trapped soluble HSPB1 increased tumor cell transendothelial migration (Figure S1A and B in File S1).

**Figure 1:**
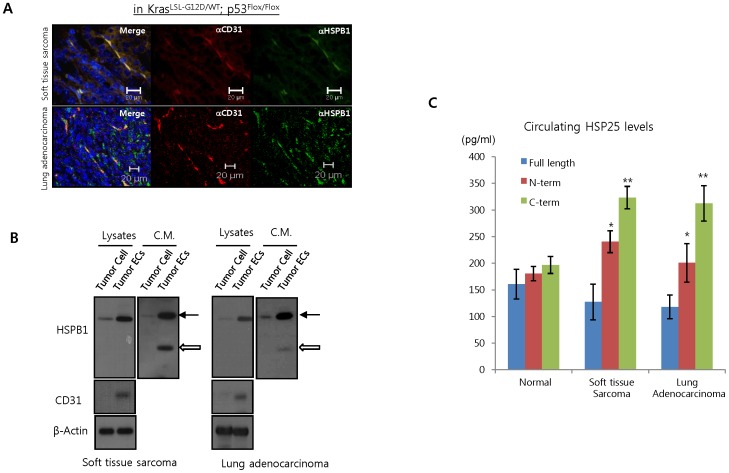
Soluble HSPB1 is secreted by tumor endothelial cells. **A** Co-localization of HSBP1 and CD31 on tumor vessels in soft tissue sarcomas and lung adenocarcinomas derived from Kras*^LSL-G12D/WT^*; p53*^Flox/Flox^* mice was detected by immunofluorescence. **B** Tumor cells and tumor endothelial cells (ECs) were isolated from sarcomas and lung adenocarcinomas of Kras*^LSL-G12D/WT^*; p53*^Flox/Flox^* mice and cell lysates were analyzed by western blot. Secreted HSPB1 was detected in conditioned media (C.M.) from these cells. arrows, full length HSPB1; open arrows, cleaved HSPB1 **C** Serum HSPB1 levels in Kras*^LSL-G12D/WT^*; p53*^Flox/Flox^* mice with or without tumors were measured by ELISA assays for detecting intact HSPB1 as described in Methods; blue bars, anti-HSPB1 (10-21)+anti-HSPB1 (158-205) antibody. Red and green bars obtained from general ELISA assays using antibodies to the N- (10-21) or C-termini (158-201) of HSPB1 (*P<0.05 and **P<0.01 vs intact HSPB1).

As previously reported [11], we determined that HSPB1 is highly expressed and secreted in tumor vascular ECs derived from primary sarcoma and lung adenocarcinoma mouse models (K-ras^LSL-G12D/WT^; p53^Flox/Flox^) compared to tumor cells (Figure 1A, B and Figure S2 in File S1). Interestingly, cleavage of HSPB1 was detected only in conditioned media from tumor vascular ECs, but not from tumor cells (Figure 1A). To confirm the cleavage of HSPB1 *in vivo*, we performed ELISA assays using N-terminal (amino acids 10–21) and C-terminal (amino acids 158–205) HSPB1 antibodies. The cleavage of HSPB1 was higher in the serum of K-ras^LSL-G12D/WT^; p53Flox^/Flox^ mice bearing soft tissue sarcomas and lung adenocarcinomas than in that of normal mice (Figure 1C). These results suggest that secretion and fragmentation of HSPB1 occurred primarily in tumor vascular ECs, not tumor cancer cells, may be related to tumor development.

### HSPB1 is Cleaved by MMPs

**Figure 2:**
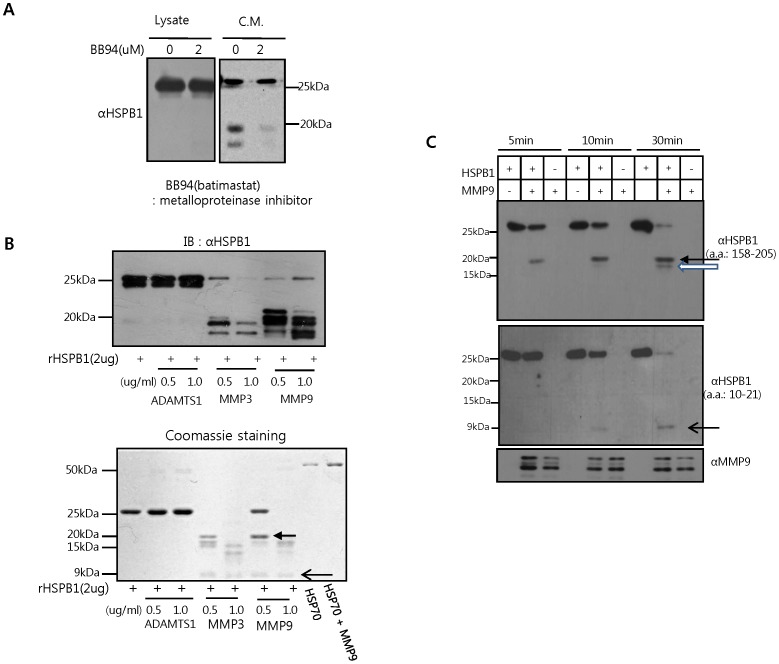
HSPB1 is cleaved by MMPs. **A** HUVECs were treated for 24(2 µM). Cleaved HSPB1 levels in conditioned media (C.M.) were detected by western blot analysis using an HSPB1 antibody to amino acids 158-205. **B** Recombinant HSPB1 (2 µg/mL) was incubated with ADAMTS1, MMP3, or MMP9 (50 ng/mL) for the indicated times. HSP70 (2 µg/mL) was incubated with MMP9 as a negative control. HSPB1 cleavage was detected by immunoblot using the HSPB1 antibody to amino acids 158-205 as well as Coomassie staining. **C** Recombinant HSPB1 (2 µg/mL) was incubated with MMP9 (50 ng/mL) for the times indicated and analyzed by western blot using antibodies against the C- (158-205) and N-termini (10–21) of HSPB1.

MMPs can cleave a variety of secreted and cell surface molecules, which regulate EC activation in pathophysiological conditions [27], [28], [29]. To test the hypothesis that HSPB1 is a substrate of MMPs, we added BB94 (an MMP inhibitor, batimastat) to HUVECs. BB94 significantly reduced HSPB1 fragment levels in HUVEC conditioned media, indicating that HSPB1 can be cleaved by MMPs (Figure 2A). In *in vitro* cleavage assays, HSPB1 was cleaved by MMP2, 3 and 9, but not by ADAMTS1, whereas HSP70 was not cleaved by MMP9 (Figure 2B, C and Figure S3 in File S1). To characterize HSPB1 fragment, we examined HSPB1 rocessing by MMP9, because MMP9 cleaved HSPB1 more clearly than MMP2 and 3 (Figure 2A and Figure S3 in File S1). Both C- and N-terminal fragments were detected after exposure to MMP9 in a time-dependent manner, indicating that cleavage occurred in at least two stages (Figure 2C). A fragment of a higher molecular mass (20 kDa) was produced first, followed by a smaller 7 kDa fragment. Besides these two major fragments, other cleavage patterns were suggested to be due to post modification such as phosphorylation or glycosylation. Interestingly, we detected the phosphorylation of fragmentation, suggesting that the lower band of 20kDa is non-phosphorylated form (Figure 2C and Figure S4 in File S1, white arrows). Also, it has been reported that O-GlcNAcylation of HSPB1 was detectable in metastatic cancer [30]. We are currently studying about post-modification patterns of fragments.

### Identification of HSPB1 Cleavage Sites

**Figure 3:**
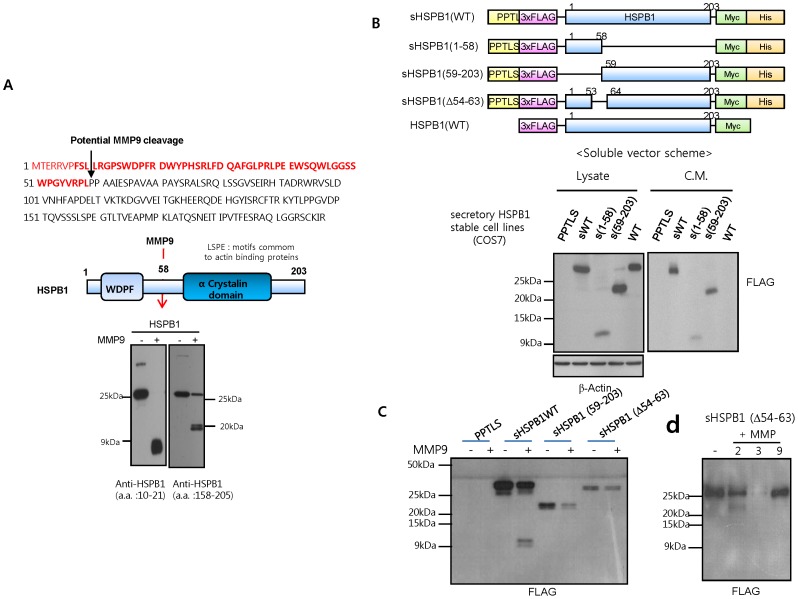
Characterization of HSPB1 intramolecular cleavage. **A** HSPB1 cleavage sites and a schematic representation of the resulting fragments. The amino acid sequence of the HSPB1 fragment from a Coomassie blue stained gel was analyzed by MALDI-TOF and LC mass spectrometry. The N-terminus or C-terminus HSPB1 fragments were detected by western blot analysis using N-terminal or C-terminal HSPB1 antibodies, respectively. **B** A schematic representation of the soluble vector for HSPB1. HSPB1 was tagged with an N-terminal 3XFlag and a C-terminal Myc tag. For secretion of HSPB1, the preprotrypsin leader sequence (PPTLS) precedes the Flag sequence; these vectors contain an additional C-terminal His tag. COS-7 cell lines stably transfected with control vector; PPTLS, secretory wild-type HSPB1; sHSPB1WT, N-terminal-deleted secretory HSPB1 (59–203); sHSPB1 (59–203), or possible cleavage sequence-deleted secretory HSPB1 (Δ54–63); sHSPB1 (Δ54–63) were cultured in OPTI-MEM media (1% FBS). Secretion of N-terminal Flag or C-terminal Myc tagged protein was detected by western blot analysis of the lysates and culture supernatants (bottom). **C** Recombinant protein (wild-type or mutant HSPB1) was incubated for 30 min with MMP9 (2 µg/mL) and HSPB1 digestion was examined by western blotting using the Flag antibody. **D** Recombinant protein of sHSPB1 (Δ54–63) was incubated for 30 min with MMP2, 3 and 9 (2 µg/mL) and HSPB1 digestion was examined by western blotting using the Flag antibody.

To identify HSPB1 cleavage sites, we performed MALDI-TOF MS and LC MS (data not shown). Results suggested that the 7 kDa fragment corresponds to the N-terminus of HSPB1 and contains the WDPF motif. Cleavage occurred between amino acids 58 and 59 of HSPB1, and the higher-molecular fragment contains the α-crystallin domain (Figure 3A). An antibody detecting amino acids 10–21 of HSPB1 specifically recognized the 7 kDa HSPB1 fragment (amino acids 1–58), whereas an antibody detecting amino acids 158–205 recognized the 20 kDa fragment (amino acids 59–205). To ensure that the fragments generated in vitro cleavage assay are the same as the ones identified in tissue culture, we used the blocking peptide against amino acid 10–21 of HSPB1 as western blot blocking buffer. The 7 kDa fragments in vitro cleavage assay and tissue culture disappeared in the presence of this blocking peptide, suggesting that the fragments generated in vitro cleavage assay are the same as the ones identified in tissue culture (Figure S5 in File S1). Taken together, these observations support the hypothesis that HSPB1 is a substrate of MMPs.

### Characterization of the Soluble HSPB1 Fragments

To further explore the significance of soluble HSPB1 cleavage, we generated secretory constructs that mimicked the MMP9-induced HSPB1 fragments (amino acids 1–58 and 59–203) as well as an MMP-resistant HSPB1 mutant (HSPB1Δ54–63; Figure 3B top). Transfection of wild-type HSPB1 that contained a secretory signal sequence (PPTLS) into COS-7 cells increased secretion of HSPB1 into the conditioned media compared to wild-type HSPB1 with no secretory signal sequence (Figure 3B, bottom). An HSPB1 mutant consisting of a 10 amino acid deletion between residues 54 and 63 did not cleaved by MMP9, indicating an MMP9-resistant form of HSPB1 (Figure 3C). MMP2 and 3 caused cleavage of this mutant, indicating that amino acids 54–63 of HSPB1 are not necessary for digestion by MMP2 and 3 (Figure 3D). Taken together, our data suggest that amino acids 54–63 of HSPB1 are accessible sites for digestion by MMP9.

### HSPB1 C-terminal Fragment Bound more Specifically to VEGF_165_

**Figure 4:**
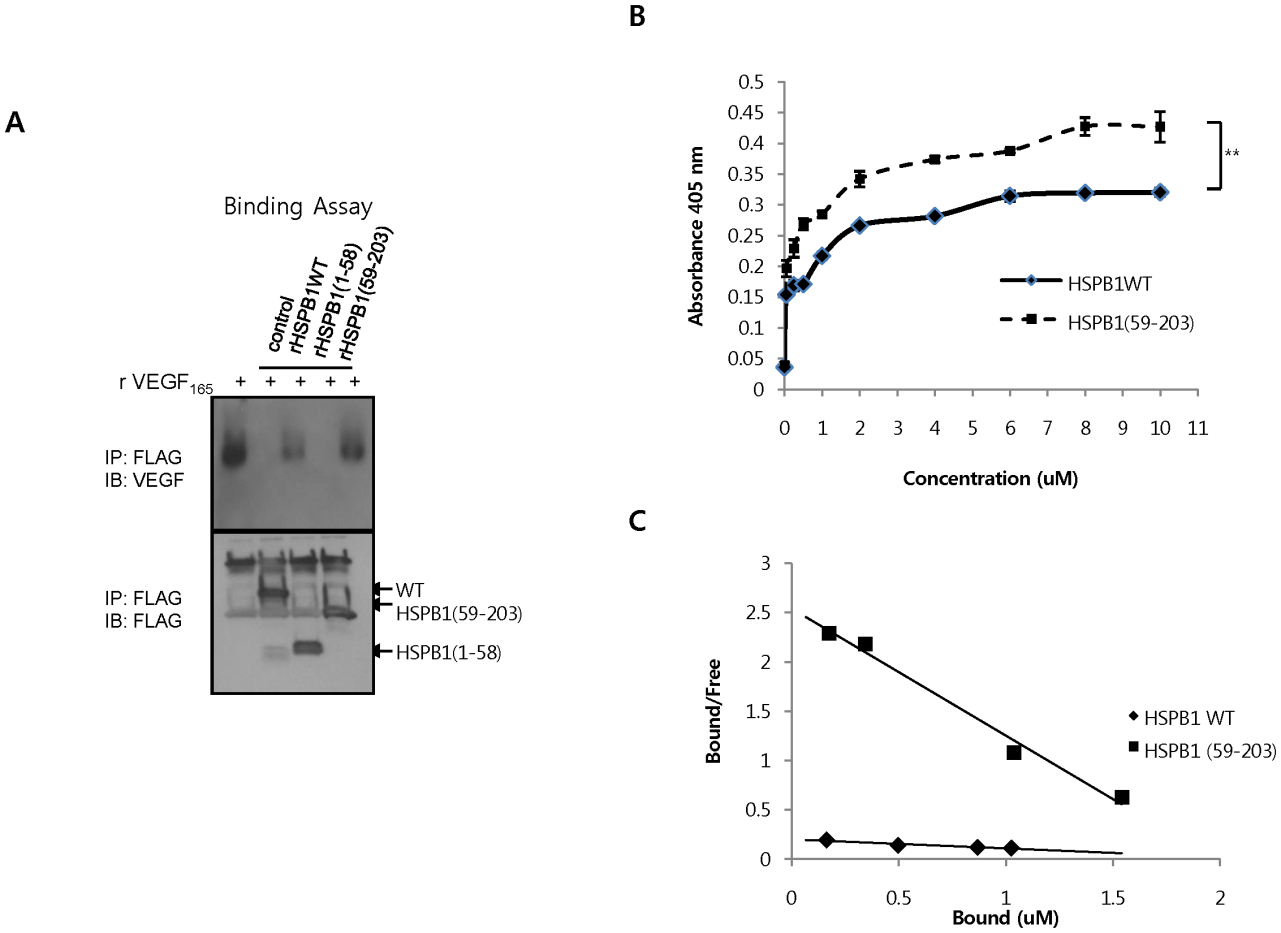
HSPB1 C-terminal fragment bound more specifically to VEGF_165_. **A** Recombinant proteins (1 µg/mL) were incubated for 6 hr at 4°C with recombinant human VEGF (50 ng/mL). Immunoprecipitates using the Flag antibody were subjected to binding assays with VEGF_165_ and western blotting using the Flag and VEGF antibodies. **B** The amount of HSPB1 WT or HSPB1 (59–203) bound to VEGF_165_ was measured by ELISA assay. Data are presented as mean ± SEM. **P <0.01. **C** K_d_ value for binding of HSPB1 WT or HSPB1 (59–203) to VEGF_165_ were calculated by Scatchard analysis. Slope of each line = − 1/K_d_.

We have previously shown that soluble HSPB1 interacts directly with VEGF**_165_** and regulates angiogenesis [31]. Thus, we investigated the binding of HSPB1 fragments to VEGF_165_. *In vitro* binding assays showed that the C-terminal fragment of HSPB1 interacted more strongly with VEGF_165_ than did wild-type HSPB1; the N-terminal fragment of HSPB1 did not interact with VEGF_165_ (Figure 4A). As shown in Figure 4a, the in vitro of VEGF**_165_**–binding activities of wild-type HSPB1 and the C-terminal fragment of HSPB1 were measured by ELISA. At a concentration range of 0.05 to 10 uM, absorbance at 405 nm (OD405) in the C-terminal fragment of HSPB1 was higher than that of HSPB1 wt, which indicates that more the C-terminal fragment of HSPB1 bound to VEGF_165_ than HSPB1 wt (Figure 4B). Using the binding curve, the dissociation constant (Kd) value of HSPB1 wt (Kd = 10.83 uM) or HSPB1 C-terminal fragment (Kd = 0.78 uM) binding to VEGF (0.5 ug) was calculated by Scatchard analysis (Figure 4C). Because a lower Kd represents a higher affinity to VEGF_165_, HSPB1 C-terminal fragment bound more specifically to VEGF.

### The MMP9-induced HSPB1 C-terminal Fragment Inhibited VEGF_165_-mediated VEGFR2 Phosphorylation

**Figure 5:**
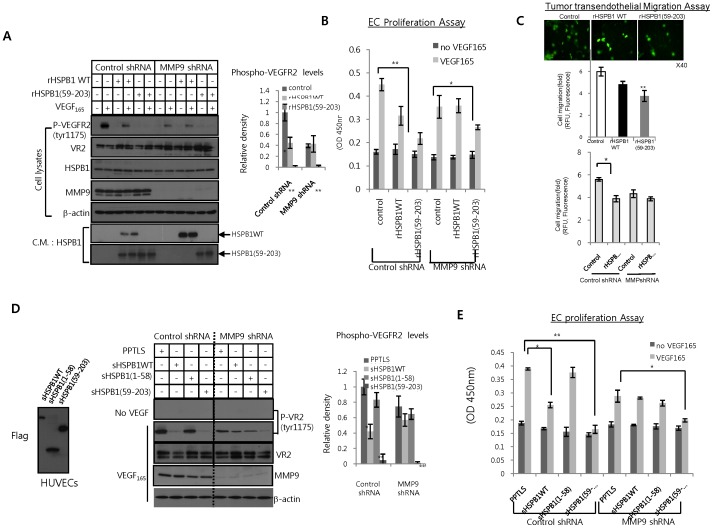
Effect of MMP9-induced HSPB1 cleavage on VEGF-mediated endothelial cell activation. **A** HUVECs infected with MMP9 shRNA lentiviral and control vectors were pretreated for 30(1ug/ml) or rHSPB1 fragments (59–203; 1 µg/ml) and then incubated with VEGF (30 ng/mL). After 10 min, cell lysates and conditioned media were subjected to western blotting using indicated antibodies. Bar graph shows ± SEM of band intensities of P-VEGFR2 signals normalized to P-VEGFR2 signals of control for 3 independent experiments. **B** After 48 hr, proliferation of HUVECs was analyzed by MTT assay. **C** Transendothelial migration of HOS cells in the presence of rHSPB1 WT (1ug/ml) or rHSPB1 fragments (59–203; 1 µg/ml). After HOS cells were marked with CytoTracker, they were allowed to attach and migrate to the HUVEC monolayer. Fluorescence graph of migratory cells labeled with CytoTracker shows ± SEM for three independent experiments (*P <0.05 and **P <0.01, upper graph). HUVECs infected with MMP9 shRNA lentiviral and control vectors were subjected to tumor transendothelial migration assay in the presence of rHSPB1 WT (1ug/ml) (bottom graph). **D** HUVECs infected with MMP9 shRNA lentiviral and control vectors were transfected with sHSPB1WT or sHSPB1 fragments (1–58, 59–203) and then activated with VEGF (30 ng/mL). After 10 min, cell lysates and conditioned media were subjected to western blotting. Bar graph shows ± SEM of band intensities of P-VEGFR2 signals normalized to P-VEGFR2 signals of control for 3 independent experiments. **E** After 48 hr, proliferation of HUVECs was analyzed by MTT assay.

To determine the effect of MMP9-cleaved HSPB1 fragments on the VEGF-VEGFR2 pathway, we knocked downMMP9 in HUVECs using MMP9 shRNA lentiviral vector. Cleavage of HSPB1 protein was significantly reduced in the conditioned media of MMP9 knock-down cells compared to control cells (Figure 5A). Consequently, in MMP9 knock-down cells, HSPB1 protein did not inhibit phosphorylation of VEGFR2 by VEGF as compared to an absence of HSPB1. Treatment with the C-terminal HSPB1 fragment was sufficient to inhibit VEGF phosphorylation (Figure 5A). Although knockdown of only MM9 in HUVECs appeared to inhibit VEGF-VEGFR2 phosphorylation, we confirmed that MMP9-cleaved HSPB1 enhanced more this inhibition (Figure 5A). These patterns of HSPB1 fragmentation regulating VEGF-VEGFR2 activity were similar to those in VEGF-induced HUVEC proliferation (Figure 5B). In addition, in control cells (PPTLS), overexpression of the C-terminal fragment of HSPB1 inhibited VEGF-VEGFR2 phosphorylation more than did wild-type HSPB1, whereas the N-terminal fragment of HSPB1 did not. In MMP9 knock-down cells, showing that control cells (PPTLS) decreased VEGF-VEGFR2 phosphorylation compared to control shRNA, wild-type HSPB1 did not inhibit VEGF-VEGFR2 phosphorylation compared to control cells (PPTLS), whereas overexpression of C-terminal HSPB1 fragment showed such inhibition (Figure 5C). These patterns were also observed in VEGF-induced HUVEC proliferation (Figure 5D). Treatment with C-terminal HSPB1 peptides inhibited VEGF-VEGFR2 activity, but N-terminal HSPB1 peptides (amino acids 10–21) did not (Figure S6 in File S1). Taken together, these observations suggest that the C-terminal fragment of HSPB1 released by MMP9 exerts greater anti-angiogenic effects than full length HSPB1.

### HSPB1 Cleavage by MMP9 Occurs during Tumor Progression

It has been previously shown that MMP9 is upregulated in tumor metastases [32], [33] and HSPB1 secretion is increased in tumorigenesis [31], [34], [35]. Thus, we investigated whether HSPB1 fragments could be detected in tumor metastases.

**Figure 6:**
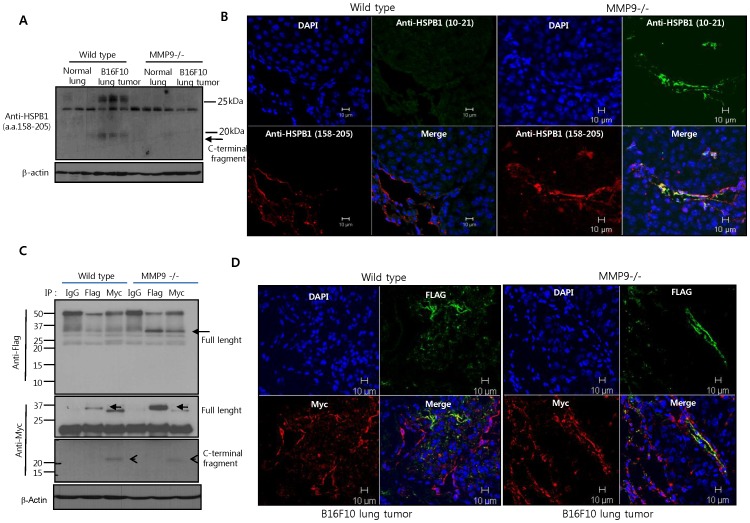
HSPB1 cleavage by MMP9 occurs during tumor progression. **A** B16F10 melanoma cell lines were injected into the tail vein of wild-type and MMP9-null mice. 14 days later, lung tumor tissues were harvested. HSPB1 levels in the lung tissue samples were analyzed by western blotting using the HSPB1 (158–205) antibody. **B** Immunofluorescence staining of the N-terminal and C-terminal HSPB1 fragments in normal and B6F10 lung tissues using the HSPB1 (10–21) and (158–205) antibodies, respectively. **C** Lung tumor tissues were obtained via intravenous injection of B6F10 cells secreting wild-type HSPB1 into wild-type or MMP9-null mice. Immunoprecipitates with control IgG, anti-Flag, and anti-Myc in lysates of lung tumor tissues were subjected to western blot analysis using the indicated antibodies. **D** Immunofluorescence staining of the N-terminal and C-terminal HSPB1 fragments in B6F10 lung tumor tissues, using the Flag and Myc antibodies, respectively.

B16F10 melanomas, which do not express HSPB1 (Fig. S7A), were injected into mice to generate lung metastases [23]. The 20 kDa fragment was observed in B16F10 lung tumor of wild-type mice but not in MMP-null mice (Figure 6A). In immunofluorescence experiments, the N- and C-terminal fragments were detected by HSPB1 antibodies recognizing amino acids 10–21 and 145–205, respectively. Co-staining with these antibodies indicated non–cleaved full length HSPB1. In lung metastases from wild-type mice, the N-terminal fragment of HSPB1 did not co-stain with the C-terminal fragment, indicating that HSPB1 cleavage occurred significantly during lung progression (Figure 6B, left). Moreover, the N-terminal HSPB1 fragment was not visible in lung tumor tissues from wild-type, suggesting that this fragment is likely degradable (Figure 6B left, Green). In fact, *in vitro* cycloheximide chase experiments exhibited that the degradation rate of the N-terminal HSPB1 fragment was significantly faster than the C-terminal fragment and wild-type HSPB1 (Figure S7B in File S1). In contrast, the N- and C-terminal fragments of HSPB1 co-localized in lung tumor tissues from MMP9-null mice, indicating that MMP9 mediates HSPB1 cleavage (Figure6B, right).

To confirm HSPB1 cleavage by MMP9 during tumor progression, we generated B6F10 cell lines secreting wild-type HSPB1 (consisting of a Flag-tagged N-terminus and a Myc-tagged C-terminus). HSPB1 secretion was confirmed by western blot analysis of conditioned media (Figure S7C in File S1). When HSPB1-secreting B6F10 cells were used to generate lung tumors, immunoprecipitation of lung tissues with the Flag or Myc antibodies pulled down full-length HSPB1 in MMP9-null samples, but not in wild-type samples (Figure 6C, top). Immunoprecipitation of lung tumor with the Myc antibody but not the Flag antibody, pulled down a 20 kDa fragment corresponding to the C-terminal wild-type HSPB1 fragment. This fragment was detected to a lesser degree in the MMP-null sample (Figure 6C, bottom, open arrows).

In addition, co-localization of Flag and Myc was more significantly detected in a MMP9-null sample than in an wild-type sample, indicating that HSPB1 cleavage reduced in MMP9-null mice (Figure 6D). A small amount of C-terminal fragment detected in MMP9 null mice is suggested due to other MMPs processing such as MMP2 and 3, eventhough overall cleavage pattern of HSBP1 by MMP2 and 3 is different from MMP9. Moreover, HSPB1 wild-type or fragments secreted by B16F10 stable cell lines were detected significantly in tumor endothelia, suggesting that soluble HSPB1 targets tumor vascular endothelium and regulates tumor vasculature (Figure 6B, D). In addition, we confirmed serum MMP9 levels of wild-type mice and MMP9-null mice using ELISA assays (Figure S8A in File S1). MMP9 secretion was increased in wild-type mice with lung tumor of B6F10 cell lines compared to normal wild-type mice, while secreted MMP9 was not detected in the serum of MMP null mice. (Figure S8A in File S1). Using ELISA assays, a double positive response of Flag or myc antibody was significantly increased in MMP9-null mice compared to wild-type mice, indicating reduced HSPB1 cleavage in the MMP9 knockout strain (Figure S8B in File S1). Taken together, our results suggest that HSPB1 is mainly cleaved by MMP9 during tumor progression.

### HSPB1 Fragments Inhibit Tumor Metastasis

**Figure 7:**
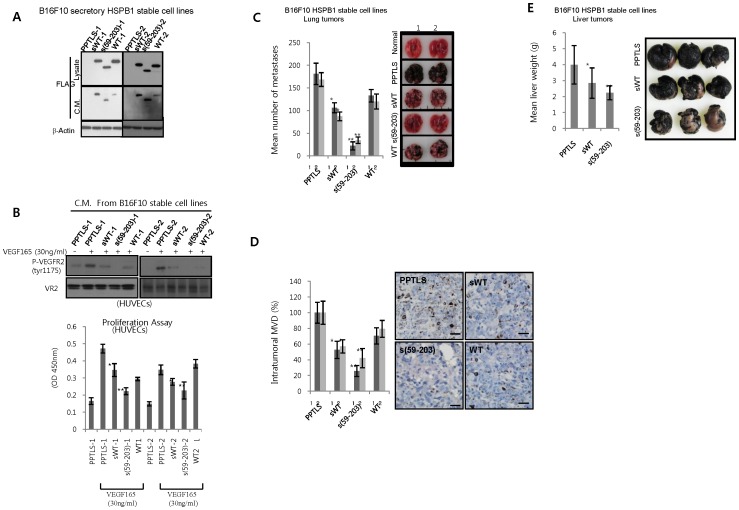
Effect of HSPB1 cleavage on tumor progression. **A** B16F10 melanoma cell lines were stably transfected with PPTLS, sHSPB1WT, sHSPB1 (59–203), and HSPB1WT. Cell lysates and conditioned media of two stable lines (1,2) were repectively subjected to western blotting (top). **B** Effects of conditioned media of B16F10 stable cell lines on VEGF_165_-induced VEGFR2 activation and proliferation activity in HUVECs. **C** Mean number of metastases at 14 days after intravenous injection of B6F10 stable cells into wild-type mice. The graph shows the mean ± SEM from three independent experiments of six mice per group f (*P <0.05 and **P <0.01 vs PPTLS). Right panel shows representative images. **D** Intratumoral microvessel density (MVD) per high-powered field (h.p.f.) was quantified by CD31 immunofluorescence. (*P <0.05 and **P <0.01 vs PPTLS). **E** Mean weight of liver with tumor at 14 days after intrasplenic injection of B6F10 stable cells into wild-type mice.

Because the C-terminal HSPB1 fragment inhibits VEGF-mediated angiogenesis, we hypothesized that HSPB1 cleavage inhibits VEGF-regulated tumor progression *in vivo*. We examined the effects of the C-terminal HSPB1 fragments on lung tumor progression by generating C-terminal HSPB1-secreting B16F10 cell lines. Secretion of N-terminal HSPB1 fragments was confirmed by western blot analysis of conditioned media (Figure 7A). Additionally, B6F10 cell lines stably transfected with secretory wild-type HSPB1 (s-WT) secreted more HSPB1 than did cell lines with non-secretory wild-type HSPB1 (WT). Conditioned media from B16 F10 cell lines transfected with secretory C-terminal HSPB1 (s-(59–203)) decreased more significantly VEGF_165_-induced VEGFR2 phosphorylation and proliferation of HUVECs than secretory wild-type HSPB1 (s-WT). These data suggest that the secreted C-terminal HSPB1 fragment was also biologically active in abrogating VEGF-mediated endothelial cell activation (Figure 7B). The *in vitro* growth rates of the stably transfected cell lines [s-WT, s-(59–203), and WT] were equivalent to the growth rate of the control cell line (Figure S9 in File S1). Secretory wild-type HSPB1 inhibited lung progression more than did the non-secretory wild-type HSPB1. Moreover, the secretory C-terminal HSPB1 fragment effectively inhibited growth of B16F10 lung tumor, as compared to wild-type secretory HSPB1 (Figure S7C in File S1). These patterns were similar to those of the microvessel density (MVD) of lung tumors. The secretory C-terminal HSPB1 fragment significantly reduced intratumoral MVD compared to secretory HSPB1 wild-type (Figure S7d in File S1).

Collectively, HSPB1 cleavage by MMP9 is inhibited in B16F10 lung tumor progression and MVD. In addition, We also examined the effect of HSPB1 fragment on liver tumor progression. Splenic injection of B16F10 cell lines with the secretory C-terminal HSPB1 effectively reduced liver tumor progression, compared to secretory wild-type HSPB1 (Figure 7e). To confirm the effect of HSPB1 fragment on other tumor cell lines, we made lung tumors using CT26 colon carcinoma cells. Secretory C-terminal HSPB1 significantly inhibited CT26 lung tumor, while secretory wild-type HSPB1 did not (Figure S10 in File S1). We suggest that the inhibitory function of secretory wild-type HSPB1 may be depend on tumor cell types, but C-terminal HSPB1 have the inhibitory function consistently. Also, these data suggest that full-length HSPB1 protein requires proteolytic degradation to release the C-terminal fragment in order to endogenously inhibit tumor progression by regulating VEGF-induced ECs activation.

## Discussion

We investigated the potential role of MMPs in the generation of bioactive HSPB1. Here, we have identified HSPB1 as a novel substrate of MMP9. Interestingly, we demonstrate that this cleavage generates a more anti-angiogenic HSPB1 fragment having endothelial inhibitory activity via interaction with VEGF. Furthermore, HSPB1 cleavage by MMP9 occurred during tumor progression and the anti-angiogenic HSPB1 fragment inhibited intratumoral MVD and tumor growth. VEGF_165_ is known to be cleaved by MMPs such as MMP9 and MMP3. Also, we found that HSPB1 bound toVEGF_121_ lesser that VEGF_165_ (Figure S11 in File S1). We are currently studying the structure of VEGF-HSPB1 complex, and this may explain the difference of binding activities. Overall, these findings suggest that generation of anti-angiogenic fragments from HSPB1 is important for understanding the physiological function of MMP9.

Recent studies have shown that serum HSPB1 levels are related to disease, suggesting it as a prognostic marker [36], [37], [38]. Serum HSPB1 was decreased in atherosclerotic patients due to proteolytic degradation in atherosclerotic plaques [39]. In addition, many studies have shown that HSPB1 is upregulated in tumor tissues [40], [41], [42]. Although increased blood serum HSPB1 levels have been observed in cancer patients, only a small number of studies have investigated the role of soluble HSPB1. Previously, we showed that soluble HSPB1 is released primarily from endothelial cells (ECs) and plays a key role in regulating the angiogenic balance via direct interaction with VEGF [11]. Here, we suggest that HSPB1 is primarily secreted from tumor endothelial cells, not tumor cells and cleavage of soluble HSPB1 by MMP9 leads to endogenous anti-angiogenic effects during tumor progression. The C-terminal HSPB1 fragment by MMP9 binds more to VEGF_165_ than full-length HSPB1, which strongly inhibits VEGF_165_-induced VEGFR2 activity on ECs. And subsequently, the reduced VEGF-induced VEGFR2 activity may decrease the proliferation of ECs and tumor cell transendothelial migration, causing decreased tumor progression. To our knowledge, this is the first study to demonstrate that HSPB1 cleavage by MMPs is associated with activity against tumor progression.

Taken together, our data suggest that soluble HSPB1 inhibits VEGF function and that cleavage of soluble HSPB1 is increased as a host defense mechanism against tumor angiogenesis. Host defenses that inhibit tumor angiogenesis include the endogenous angiogenesis inhibitors thrombospondin-1 and endostatin, which counteract the effects of pro-angiogenic factors. This function prevents the angiogenic switch in *in situ* carcinomas, which inhibits the transition to an angiogenic phenotype [43], [44], [45], [46]. In fact, the matrix metalloproteinase ADAMTS1 mediates the release of anti-angiogenic fragments from TSP1 and 2 [23]. MMPs produce endostatin, an inhibitor of angiogenesis, from collagen XVIII [18]. In addition, we found that in PMA-treated U937 cells, HSPB1 cleavage occurred depending on MMP9 (Figure S12 in File S1), suggesting that in tumor microenvironment, macrophages play a role in MMP9-induced HSPB1 cleavage.

Our data demonstrate that the C-terminal HSPB1 fragment inhibits VEGF-induced angiogenesis to a greater extent than does full-length HSPB1. Additionally, the N-terminal fragment was detected to a lesser degree in tumor metastatic tissues and blood than the C-terminal fragment, suggesting that this fragment is likely more degraded (Figure 6 and Figure S8b in File S1). Indeed, *in vitro* cycloheximide chase experiments supported that the degradation rate of the N-terminal HSPB1 fragment was significantly faster than the C-terminal fragment and wild-type HSPB1 (Figure S7b in File S1). Also, it was recently reported that the N-terminus of HSPB1 is flexible and unfolded. Therefore, we hypothesize that increased MMP9 activity during tumorprogression. induces HSPB1 cleavage, and subsequently, cleaved C-terminal HSPB1 interacts with VEGF more than intact HSPB1 due to conformational changes. As shown inFigure 6, wild type and C-terminal fragment of HSPB1 were mainly detected in tumor endothelium during tumor progression, suggesting that secreted and cleaved HSPB1 targeting tumor endothelium may play an important role in the regulation of tumor angiogenesis. We are currently studying the effects of HSPB1 glycosylation, phosphorylation and dimerization on MMP9-induced HSPB1 cleavage during tumor progression. Taken together, our data suggest that MMP9-induced HSPB1 cleavage acts to regulate the activity of -tumor vascular ECs, releasing anti-angiogenic C-terminal fragment against tumor progression.

In clinical trials of MMP inhibitors (MMPI) as anticancer drugs, MMPIs failed to control advanced cancer. Some reports indicate that this was due to the loss of the beneficial and protective actions of MMP anti-targets that counterbalance the benefits of therapeutically blocking MMP targets in cancer [16], [47], [48], [49]. It has been suggested that the anti-angiogenic properties of MMPs may be one of the most critical MMP anti-target activities [18], [50]. In this study, we suggest that MMPIs destroy the biological processes underlying the maintenance of an endogenous anti-angiogenic balance, leading to the failed clinical trials. Finally, HSPB1 fragment levels in the blood of cancer patients may be a valuable prognostic marker of MMPIs. Development of novel MMP substrates may thus provide new strategies for successful cancer therapies based on MMP inhibition.

## Supporting Information

File S1 **Contains the following: Figure S1. The effect of soluble HSPB1 on tumor transendothelial migration** A. Transendothelial migration of HOS cells in the presence of HSPB1 or an HSPB1-neutralizing antibody. HUVEC monolayers were treated with PBS, control IgG, recombinant HSPB1 (1.5, 3 µg/mL), or HSPB1 neutralizing antibodies (1, 2 µg/mL). After HOS cells were marked with CytoTracker, they were allowed to attach and migrate to the HUVEC monolayer. Fluorescence graph of migratory cells labeled with CytoTracker shows ± SEM for three independent experiments (*P<0.05 and **P<0.01). B. Neutralizing activity of the HSP27 antibodies (Stressgene) was assessed in C.M. from HUVECs. HSP27 antibodies (1ug/ml) were incubated with 3 ml of C.M. for 6 hr and the immune complexes precipitated with protein A-Sepharose (80uL; Sigma) and analyzed by SDS-PAGE. **Figure S2. Localization of HSPB1 in cancer tissue.** Immunohistochemical analyses of HSPB1 in soft tissue sarcomas and lung adenocarcinomas derived from KrasLSL-G12D/WT; p53Flox/Flox mice using antibody against HSPB1 C-terminus (Santa cruz). (size bar, 20ul) **Figure S3. HSPB1 cleavage by MMP2 or MMP3.** Recombinant HSPB1 (2 µg/mL) was incubated with MMP2 and 3 (50 ng/mL) for the indicated times and analyzed by western blot using antibodies against the C- (158–205) and N-termini (10–21) of HSPB1. **Figure S4. Phosphorylation patterns of HSPB1 fragments**. HUVECs cell lysates were incubated with MMP9 (50 ng/mL) for 30 min and analyzed by western blot using antibodies against HSPB1, phospho-HSPB1 (Ser82, Stressgene) and phospho-HSPB1 (Ser72, Stressgene). **Figure S5. Identification of HSPB1 fragment by blocking peptide.** HUVECs lysates incubated with MMP9 (50 ng/mL) for 30 min and conditioned media after 2 days incubation with Opti-MEM (0.1% FBS) were subjected to western blot using mixed antibodies against HSPB1 (10–21)+HSPB1 (158–205) in the presence of blocking peptide against amino acid 10–21 of HSPB1 or not. **Figure S6. The effect of N-terminal (10–21) or C-terminal HSPB1 peptide on VEGF-VEGFR2 activity.** HUVECs were pretreated with N-terminal (10–21) or C-terminal HSPB1 peptide (Abcam) for 30 min. After 5 min stimulation of HUVECs with VEGF, the VEGFR2 phosphorylation status was detected by western blotting. **Figure S7. The degradation rate of the HSPB1 fragments** A. HSPB1 expression level in B16 F10 melanoma cells was determined by western blotting. B. COS-7 cell lines expressing N-terminal Flag tagged secretory wild-type HSPB1, N-terminal HSPB1 (1–58) or C-terminal HSPB1 (59–203) were treated with cycloheximide (CHX, Sigma). After indicated time points, Flag tagged protein in conditioned media was detected by western blot analysis. C.Transfection efficacy of secretary HSPB1 wild type in B16 F10 melanoma cells was determined by western blotting. **Figure S8. A. Serum MMP9 levels were analyzed by ELISA.** Serum MMP9 of wild-type and MMP9-null mice bearing B16F10 melanoma lung metastic tumor was analyzed by ELISA. B. HSPB1 fragments in serum were examined by ELISA for intact HSPB1 using Flag and Myc antibodies as described in Methods; blue bars (Intact HSPB1), anti-Flag (N-terminus)+anti-Myc (C-terminus) antibody. Red (N-terminus fragment) and green (C-terminus fragment) bars obtained from general ELISA assays using antibodies to the Flag (N-terminus) or Myc (C-terminus) of HSPB1 (*P<0.05 and **P<0.01 vs intact HSPB1). **Figure S9. The growth rates of stably transfected B16F10 cells were measured by MTT assay.** **Figure S10. The effect of HSPB1 cleavage on lung tumor progression of CT26 colon carcinoma cells** A. CT26 colon carcinoma cell lines were stably transfected with PPTLS, sHSPB1WT, sHSPB1 (59–203). Cell lysates and conditioned media were subjected to western blotting (top). B. Mean weight of lung tumor at 14 days after intravenous injection of CT26 stable cells into wild-type mice. The graph shows the mean ± SEM from three independent experiments of six mice per group f (**P<0.01 vs sWT). Right panel shows representative images. **Figure S11. HSPB1 bound slightly to VEGF121.** Flag-tagged recombinant HSPB1 Wild type proteins (1 µg/mL) were incubated for 6 hr at 4°C with recombinant human VEGF121 or VEGF165 (50 ng/mL). Immunoprecipitates using the Flag antibodies were subjected to binding assays with VEGF121 orVEGF165 and western blotting using the Flag and VEGF antibodies (R&D). **Figure S12. MMP9- dependent HSPB1 cleavage in PMA-treated U937 cells** PMA (20ng/ml, 24 hr) –treated U937 cells were infected with MMP9 shRNA lentiviral and control vectors, and then were subjected to HSPB1 cleavage assay in vitro.
(PDF)
