# Supplementary material for: MMP9 Processing of HSPB1 Regulates Tumor Progression
Source: PLoS One. 2014 Jan 20;9(1):e85509. doi: 10.1371/journal.pone.0085509 (PMC3896397; doi:10.1371/journal.pone.0085509)
Supplement: File S1 — Contains the following: Figure S1. The effect of soluble HSPB1 on tumor transendothelial migration A. Transendothelial migration of HOS cells in the presence of HSPB1 or an HSPB1-neutralizing antibody. HUVEC monolayers were treated with PBS, control IgG, recombinant HSPB1 (1.5, 3 µg/mL), or HSPB1 neutralizing antibodies (1, 2 µg/mL). After HOS cells were marked with CytoTracker, they were allowed to attach and migrate to the HUVEC monolayer. Fluorescence graph of migratory cells labeled with CytoTracker shows ± SEM for three independent experiments (*P<0.05 and **P<0.01). B. Neutralizing activity of the HSP27 antibodies (Stressgene) was assessed in C.M. from HUVECs. HSP27 antibodies (1ug/ml) were incubated with 3 ml of C.M. for 6 hr and the immune complexes precipitated with protein A-Sepharose (80uL; Sigma) and analyzed by SDS-PAGE. Figure S2. Localization of HSPB1 in cancer tissue. Immunohistochemical analyses of HSPB1 in soft tissue sarcomas and lung adenocarcinomas derived from KrasLSL-G12D/WT; p53Flox/Flox mice using antibody against HSPB1 C-terminus (Santa cruz). (size bar, 20ul) Figure S3. HSPB1 cleavage by MMP2 or MMP3. Recombinant HSPB1 (2 µg/mL) was incubated with MMP2 and 3 (50 ng/mL) for the indicated times and analyzed by western blot using antibodies against the C- (158–205) and N-termini (10–21) of HSPB1. Figure S4. Phosphorylation patterns of HSPB1 fragments. HUVECs cell lysates were incubated with MMP9 (50 ng/mL) for 30 min and analyzed by western blot using antibodies against HSPB1, phospho-HSPB1 (Ser82, Stressgene) and phospho-HSPB1 (Ser72, Stressgene). Figure S5. Identification of HSPB1 fragment by blocking peptide. HUVECs lysates incubated with MMP9 (50 ng/mL) for 30 min and conditioned media after 2 days incubation with Opti-MEM (0.1% FBS) were subjected to western blot using mixed antibodies against HSPB1 (10–21)+HSPB1 (158–205) in the presence of blocking peptide against amino acid 10–21 of HSPB1 or not. Figure S6. The effect of N-terminal (1 [file pone.0085509.s001.pdf]

Supporting Information Legends

Figure S1. **The effect of soluble HSPB1 on tumor transendothelial migration**

A. Transendothelial migration of HOS cells in the presence of HSPB1 or an HSPB1-neutralizing antibody. HUVEC monolayers were treated with PBS, control IgG, recombinant HSPB1 (1.5, 3 μg/mL), or HSPB1 neutralizing antibodies (1, 2 μg/mL). After HOS cells were marked with CytoTracker, they were allowed to attach and migrate to the HUVEC monolayer. Fluorescence graph of migratory cells labeled with CytoTracker shows ± SEM for three independent experiments ($*P<0.05$ and $**P<0.01$). B. Neutralizing activity of the HSP27 antibodies (Stressgene) was assessed in C.M. from HUVECs. HSP27 antibodies (1ug/ml) were incubated with 3 ml of C.M. for 6 hr and the immune complexes precipitated with protein A-Sepharose (80uL; Sigma) and analyzed by SDS-PAGE.

Figure S2. **Localization of HSPB1 in cancer tissue.** Immunohistochemical analyses of HSPB1 in soft tissue sarcomas and lung adenocarcinomas derived from Kras$^{LSL\text{-}G12D/WT}$; p53$^{Flox/Flox}$ mice using antibody against HSPB1 C-terminus (Santa cruz) . (size bar, 20ul)

Figure S3. **HSPB1 cleavage by MMP2 or MMP3.** Recombinant HSPB1 (2 μg/mL) was incubated with MMP2 and 3 (50 ng/mL) for the indicated times and analyzed by western blot using antibodies against the C- (158-205) and N-termini (10-21) of HSPB1

Figure S4. **Phosphorylation patterns of HSPB1 fragments.** HUVECs cell lysates were incubated with MMP9 (50 ng/mL) for 30min and analyzed by western blot using antibodies against HSPB1, phospho-HSPB1 (Ser82, Stressgene) and phospho-HSPB1 (Ser72, Stressgene).

Figure S5. Identification of HSPB1 fragment by blocking peptide. HUVECs lysates incubated with MMP9 (50 ng/mL) for 30min and conditioned media after 2 days incubation with Opti-

MEM (0.1% FBS) were subjected to western blot using mixed antibodies against HSPB1 (10-21) + HSPB1 (158-205) in the presence of blocking peptide against amino acid 10-21 of HSPB1 or not.

Figure S6. The effect of N-terminal (10-21) or C-terminal HSPB1 peptide on VEGF-VEGFR2 activity. HUVECs were pretreated with N-terminal (10-21) or C-terminal HSPB1 peptide (Abcam) for 30 min. After 5 min stimulation of HUVECs with VEGF, the VEGFR2 phosphorylation status was detected by western blotting.

Figure S7. The degradation rate of the HSPB1 fragments A. HSPB1 expression level in B16 F10 melanoma cells was determined by western blotting. B. COS-7 cell lines expressing N-terminal Flag tagged secretory wild-type HSPB1, N-terminal HSPB1 (1-58) or C-terminal HSPB1 (59-203) were treated with cycloheximide (CHX, Sigma). After indicated time points, Flag tagged protein in conditioned media was detected by western blot analysis. C.Transfection efficacy of secretary HSPB1 wild type in B16 F10 melanoma cells was determined by western blotting .

Figure S8. **A. Serum MMP9 levels were analyzed by ELISA**. Serum MMP9 of wild-type and MMP9-null mice bearing B16F10 melanoma lung metastic tumor was analyzed by ELISA. B. HSPB1 fragments in serum were examined by ELISA for intact HSPB1 using Flag and Myc antibodies as described in Methods ; blue bars (Intact HSPB1), anti-Flag (N-terminus) + anti-Myc (C-terminus) antibody. Red (N-terminus fragment) and green (C-terminus fragment) bars obtained from general ELISA assays using antibodies to the Flag (N-terminus) or Myc (C-terminus) of HSPB1 (*P <0.05 and **P <0.01 vs intact HSPB1).

Figure S9. The growth rates of stably transfected B16F10 cells were measured by MTT assay.

Figure S10. The effect of HSPB1 cleavage on lung tumor progression of CT26 colon carcinoma cells A. CT26 colon carcinoma cell lines were stably transfected with PPTLS, sHSPB1WT, sHSPB1 (59-203). Cell lysates and conditioned media were subjected to western blotting (top). B. Mean weight of lung tumor at 14 days after intravenous injection of CT26 stable cells into wild-type mice. The graph shows the mean ± SEM from three independent experiments of six mice per group f (***P* <0.01 vs sWT). Right panel shows representative images.

Figure S11. HSPB1 bound slightly to $VEGF_{121}$. Flag-tagged recombinant HSPB1 Wild type proteins (1 μg/mL) were incubated for 6 hr at 4℃ with recombinant human $VEGF_{121}$ or $VEGF_{165}$ (50 ng/mL). Immunoprecipitates using the Flag antibodies were subjected to binding assays with $VEGF_{121}$ or $VEGF_{165}$ and western blotting using the Flag and VEGF antibodies (R&D).

Figure S12. MMP9- dependent HSPB1 cleavage in PMA-treated U937 cells

PMA (20ng/ml, 24hr) –treated U937 cells were infected with MMP9 shRNA lentiviral and control vectors, and then were subjected to HSPB1 cleavage assay *in vitro*.

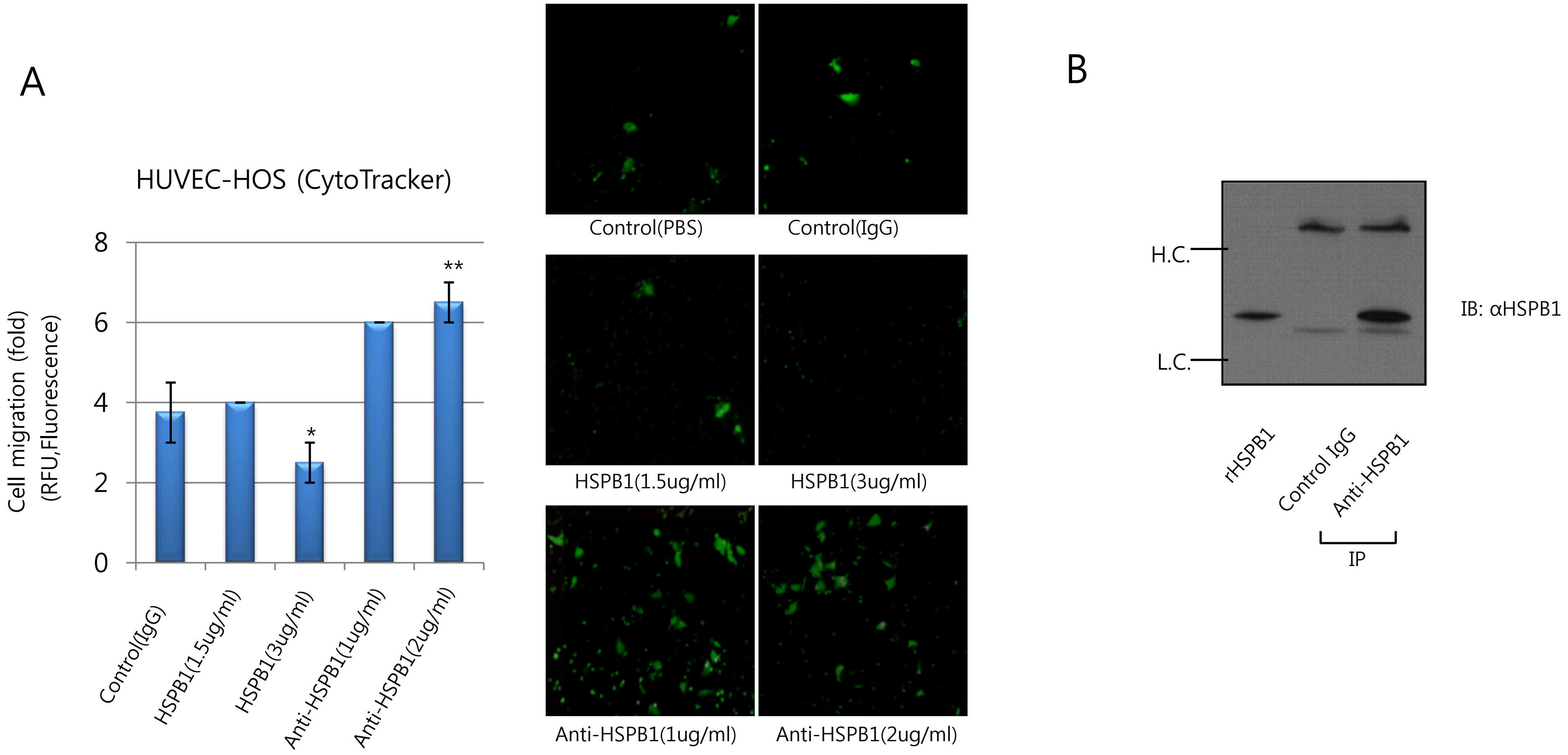

Figure S1. The effect of soluble HSPB1 on tumor transendothelial migration.

in KrasLSL-G12D/WT; p53Flox/Flox
Soft tissue sarcoma

in KrasLSL-G12D/WT; p53Flox/Flox
Lung adenocarcinoma

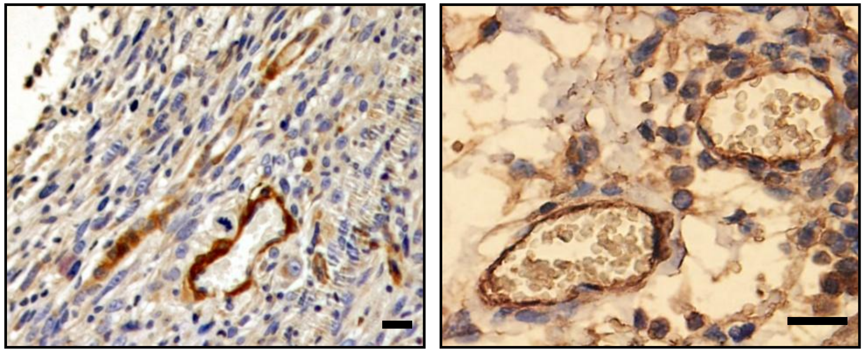

IH: HSPB1

IH: HSPB1

Figure S2. Localization of HSPB1 in cancer tissue.

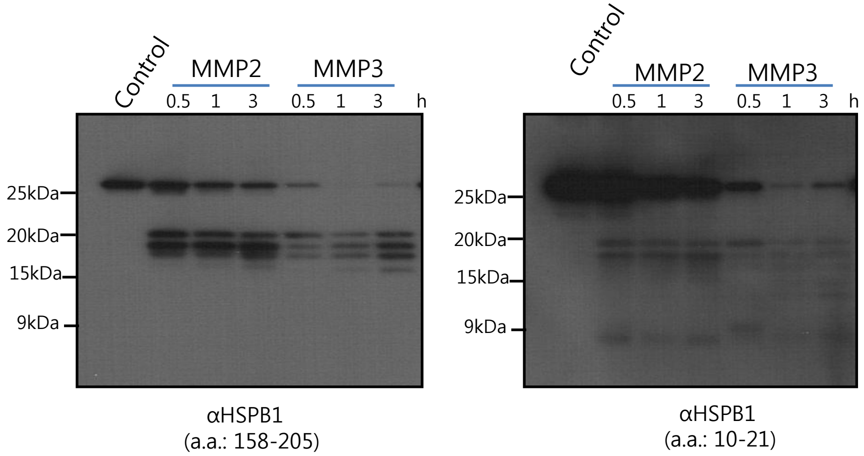

Figure S3. HSPB1 cleavage by MMP2 or MMP3.

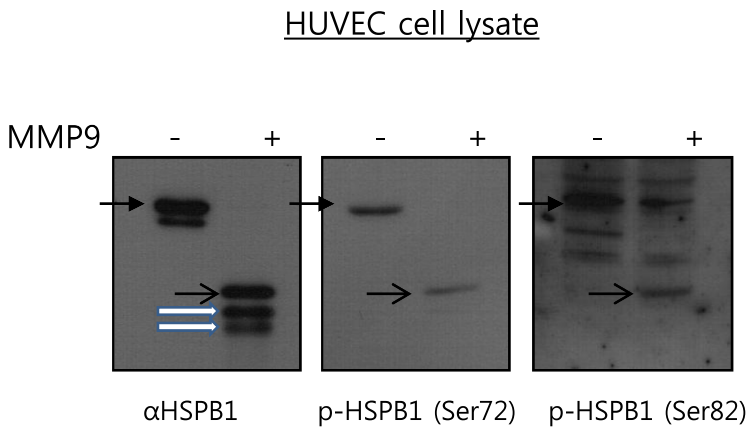

Figure S4. **Phosphorylation patterns of HSPB1 fragments.**

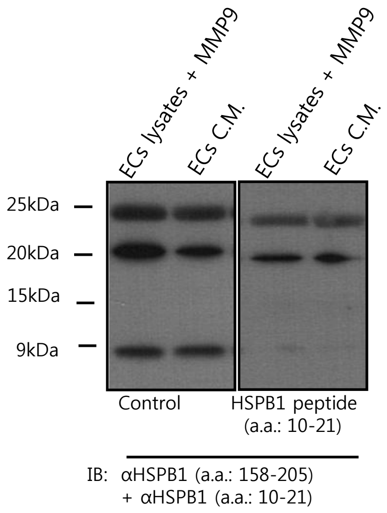

Figure S5. Identification of HSPB1 fragment by blocking peptide.

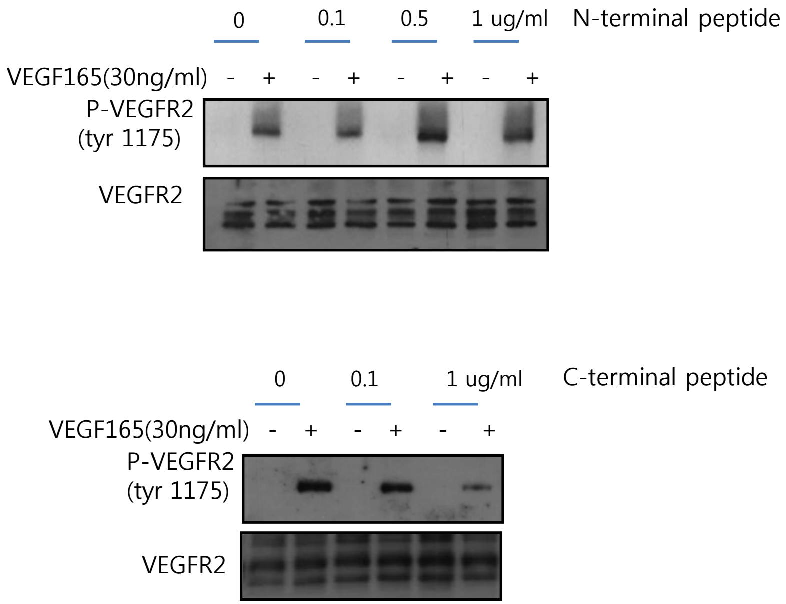

Figure S6. The effect of N-terminal (10-21) or C-terminal HSPB1 peptide on VEGF-VEGFR2 activity.

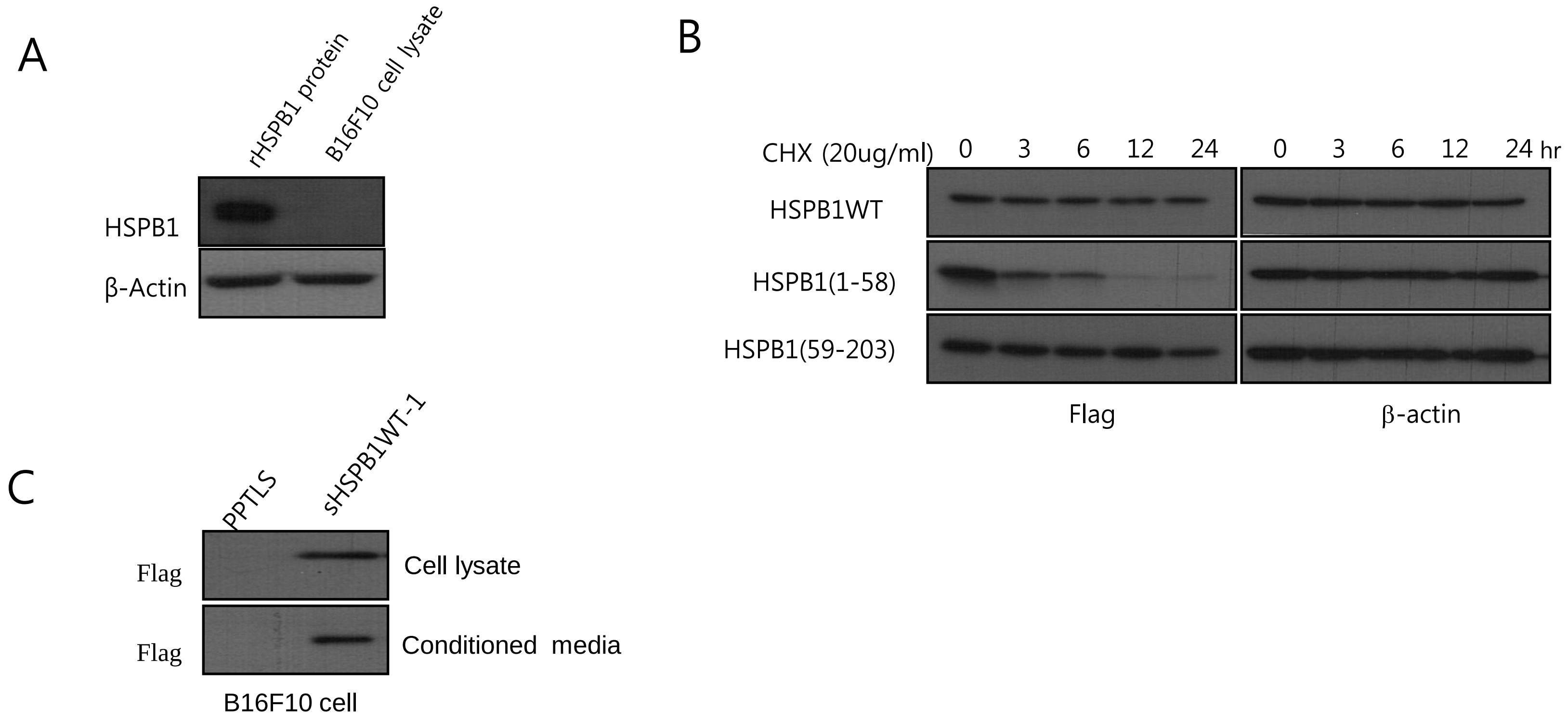

Figure S7. The degradation rate of the HSPB1 fragments.

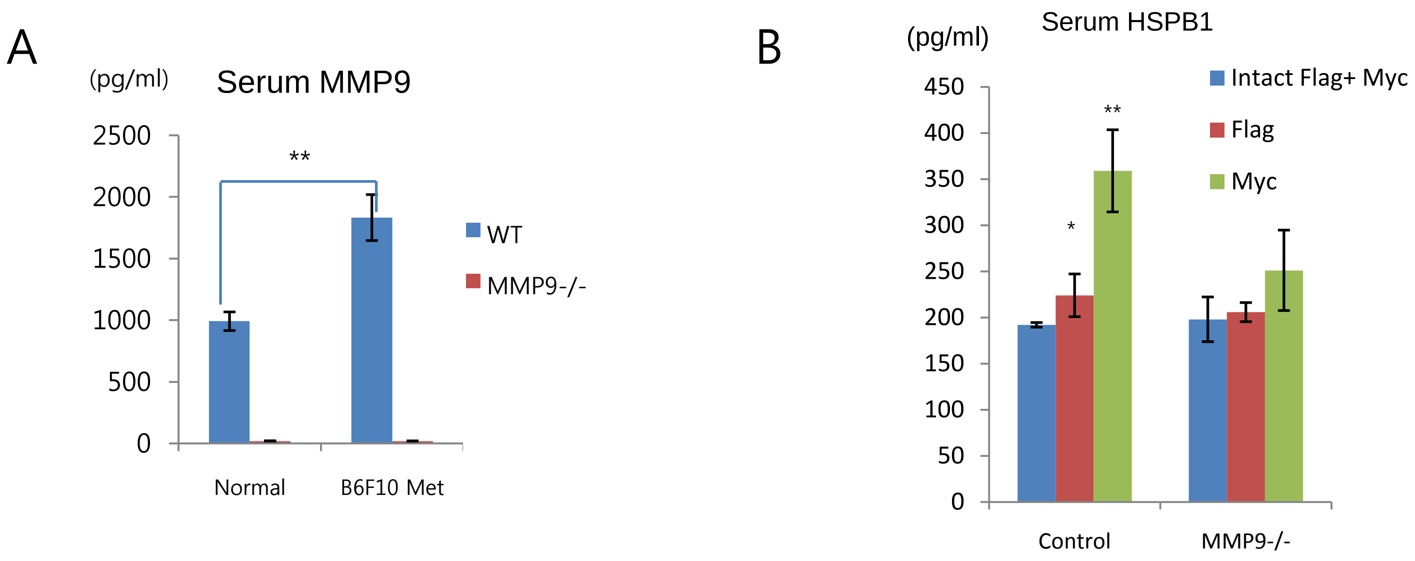

Figure S8. A. Serum MMP9 levels were analyzed by ELISA.

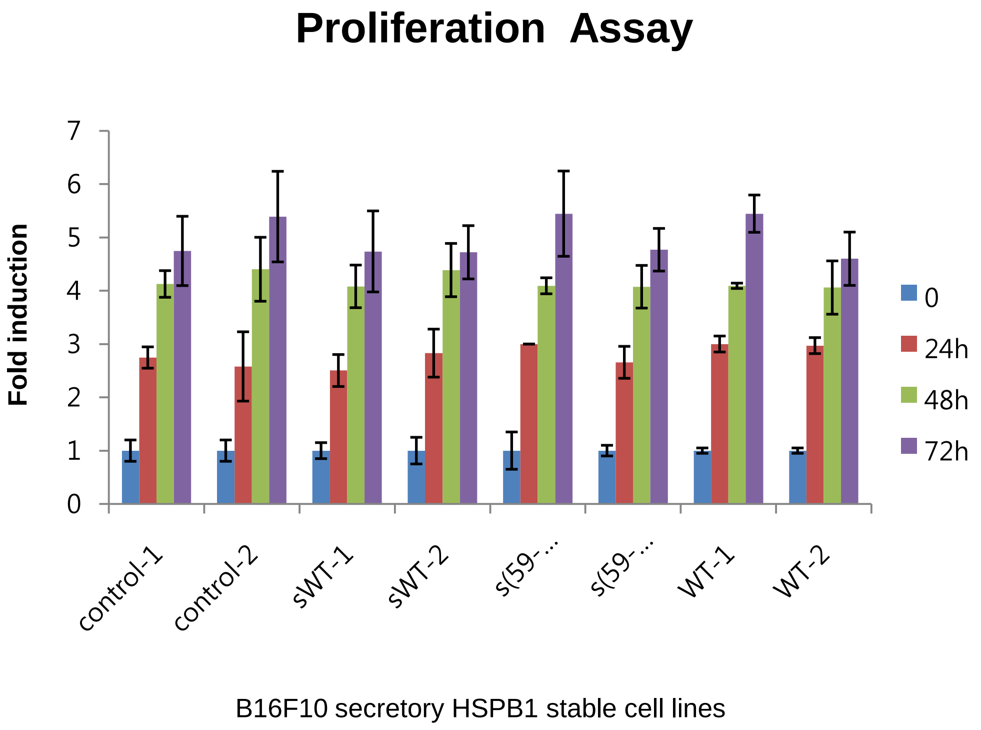

Figure S9. The growth rates of stably transfected B16F10 cells were measured by MTT assay.

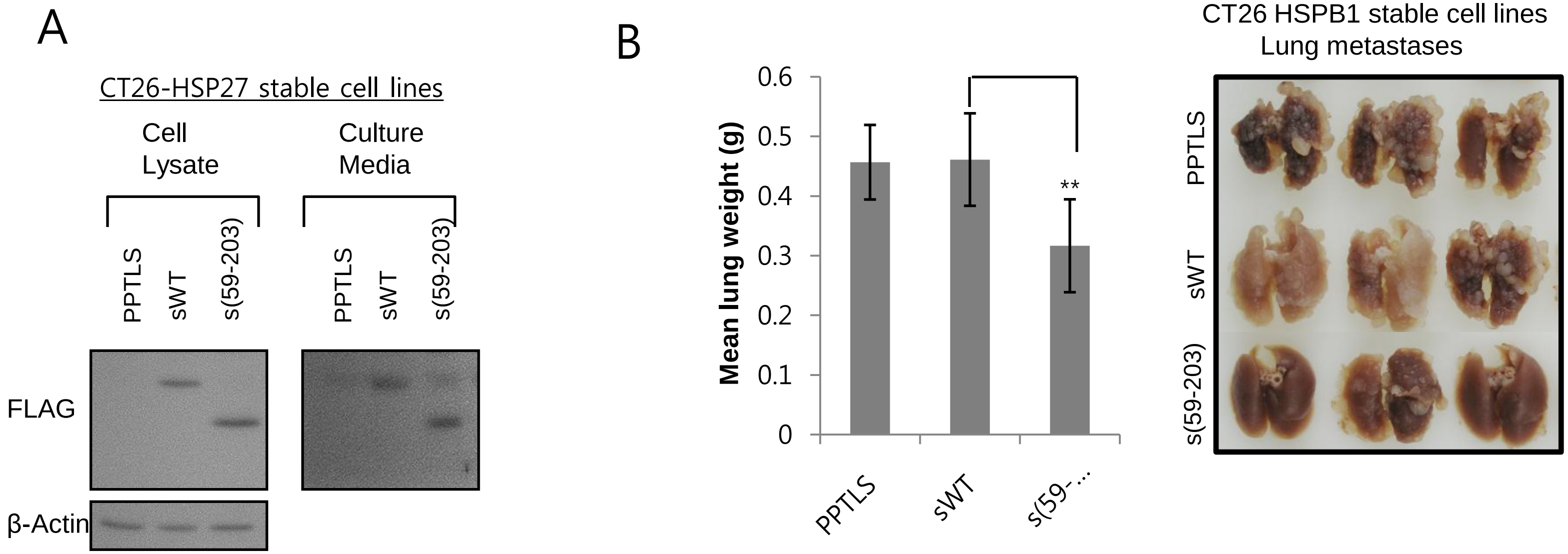

Figure S10. The effect of HSPB1 cleavage on lung tumor progression of CT26 colon carcinoma cells.

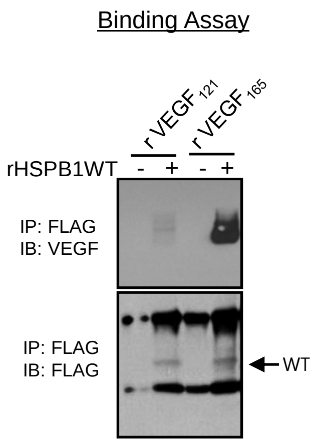

Figure S11. HSPB1 bound slightly to $VEGF_{121}$.

Cleavage Assay

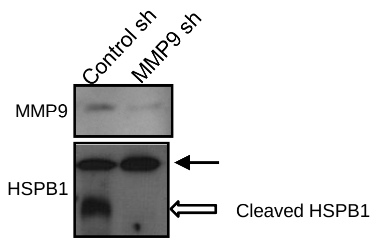

Figure S12. MMP9- dependent HSPB1 cleavage in PMA-treated U937 cells.
